# Enhancing caregiver resilience through nursing interventions in intellectual disability care-mixed method approach

**DOI:** 10.6026/973206300212728

**Published:** 2025-08-31

**Authors:** Vijayalakshmi Arumugam, Venkatesh Mathan Kumar Vasudevan, Shankar Shanmugam Rajendran, Marudan Anbalagan, Srividhya Balasubramanyan, Senthilkumar Azhagirisamy, Maheswari Narayanasamy

**Affiliations:** 1 Department of Mental Health Nursing, College of Nursing, Madras Medical College, The Tamil Nadu Dr.MGR Medical University, Chennai, Tamil Nadu, India; 2 Department of Psychiatry, Institute of Mental Health, Madras Medical College, The Tamil Nadu Dr.MGR Medical University, Chennai, Tamil Nadu, India; 3 Department of Child Health Nursing, College of nursing, Madras medical college, The Tamil Nadu Dr.MGR Medical University, Chennai, Tamil Nadu, India

**Keywords:** Care expectations, psychological distress, strain, primary care givers, intellectual disability, nurse led intervention

## Abstract

The care expectations and psychological impact on primary caregivers of individuals with intellectual disabilities, focusing on a nurse-led intervention with psychotherapy. Using a mixed-methods approach, qualitative data were collected from 5 caregivers and quantitative data from 60 caregivers over a four-week period. The intervention significantly reduced caregiver distress and strain, with improvements in psychological well-being and coping strategies. Six key themes emerged from the qualitative phase, indicating emotional relief and enhanced caregiving ability. Thus, we show the importance of structured interventions for supporting caregivers' mental health.

## Background:

Caring for individuals with intellectual disabilities (ID) is a profound and multifaceted responsibility that requires a deep commitment of time, energy and emotional resources [1]. Primary caregivers, who are often family members, play an essential role in ensuring that individuals with intellectual disabilities receive the necessary care, attention and support for their well-being [2]. This caregiving responsibility, while an expression of love and dedication, can be exceptionally challenging. Caregivers often navigate a complex and demanding journey, which includes attending to the physical, emotional and social needs of their loved ones while managing the significant strain that caregiving places on their own lives [3]. Intellectual disabilities encompass a wide range of conditions, often characterized by limitations in cognitive functioning and adaptive behaviors [4]. These conditions may vary in severity, but the common denominator among all individuals with ID is the need for consistent and specialized care. This is where primary caregivers, typically family members, step in to provide support [5]. In addition to the physical care involved, such as ensuring proper nutrition, medication and medical appointments, caregivers also play a pivotal role in promoting emotional stability and facilitating social integration for individuals with intellectual disabilities [6]. The emotional and social well-being of those with ID is largely contingent on the dedication and psychological resilience of their caregivers [7]. However, the impact of caregiving extends beyond the care recipient and affects the caregivers themselves. Many caregivers experience significant distress due to the continuous demands of care [8]. These demands often lead to caregiver strain, which includes feelings of being overwhelmed, fatigued, or helpless and can exacerbate mental health issues such as anxiety, depression and stress. Studies have shown that caregivers of individuals with intellectual disabilities are at a heightened risk of experiencing poor mental health outcomes due to the prolonged nature of caregiving and the emotional toll it takes [9]. Unfortunately, caregivers' needs are often overlooked and their emotional and psychological well-being may not be addressed adequately in healthcare settings [10]. This gap in caregiver support underscores the importance of developing structured interventions that address both the physical and emotional needs of caregivers. A nurse-led intervention incorporating psychotherapy is one such approach that could potentially alleviate caregiver strain and improve their psychological well-being [11]. The current study seeks to explore the care expectations and psychological impact on primary caregivers, specifically evaluating the effectiveness of a nurse-led intervention that includes psychotherapy in reducing distress and strain. By investigating the experiences of caregivers and measuring the psychological outcomes before and after the intervention. Therefore, it is of interest to provide valuable insights into how targeted interventions can support caregivers and enhance their coping strategies.

## Methodology:

This study employed a mixed-method approach, specifically an exploratory sequential design, to evaluate the care expectations and nurse-led interventions on psychological distress and strain among primary caregivers of children with intellectual disabilities. The qualitative component utilized a phenomenological methodology to thoroughly investigate the care experiences of primary caregivers of intellectually disabled children. These themes reflect the multifaceted experiences of caregivers. Using a one-group pre-test and post-test design, the quantitative component assessed the psychological distress and strain of caregivers at a tertiary care hospital in Chennai. This facilitated the gathering of detailed and vivid narratives from five primary caregivers regarding their expectations before the intervention. Participation in this phase was limited to sixty elderly individuals. Data were gathered using the Kessler Psychological Distress Scale and the Caregiver Strain Index. Both research components were carried out over four weeks. The intervention included organized activities focused on understanding parent, adult, and child ego states to regulate emotional responses, identifying stressors, effective communication, stress coping strategies, self-care, and emotional regulation, as well as encouraging relaxation techniques and boundary-setting to prevent burnout. The quantitative phase data were analyzed using SPSS version 22. McNemar's test and paired t-tests were employed to establish statistical significance, while thematic analysis was used to explain the qualitative data. The study obtained clearance from the Institutional Ethics Committee of Madras Medical College (Approval No. IEC-MMC/59112024), dated 19/11/2024, in Chennai, to comply with ethical norms.

## Results:

Table 1, 2 (see PDF) shows 30% of the primary caregivers are below 30 years of age, 16.67% are between 31-35 years, 33.33% fall in the 36-40 years range and 20% are aged 41-45 years.60% of the caregivers are female, while 40% are male. Among caregivers, 41.67% identify as Hindu, 25% as Christian, 15% as Muslim and 18.33% belong to other religions. Family types include 41.67% from nuclear families, 36.67% from joint families and 21.66% from extended families. Residential areas are distributed as 38.33% urban, 46.67% semiurban and 15% rural. Two-thirds (66.67%) of the caregivers are in consanguineous marriages, with 33.33% in non-consanguineous marriages. Educational qualifications show 13.33% with informal education, 26.67% with primary education, 20% completed higher secondary school, 23.33% are graduates and 16.67% hold professional qualifications. Occupational status includes 8.33% homemakers, 25% unskilled workers, 18.33% clerks/shop owners/fathers, 28.34% semi-professionals and 20% professionals. Monthly family income distribution reveals 38.33% earn below ₹5,000, 25% earn between ₹5,000 and ₹10,000 and 36.67% earn more than ₹10,000. 26.67% of the children are aged between 1-5 years, 20% are between 6-10 years and the majority, 53.33%, fall within the 11-15 years age group.51.67% of the children are male, while 48.33% are female.46.67% of the children are first-born, 25% are second-born and 28.33% are third-born or beyond.45% of the families have one child, 13.33% have two children and 41.67% have three or more children. 10% of the children were diagnosed within the last 6 months to 1 year, 40% received their diagnosis between 1-3 years ago, 36.67% between 3-5 years ago and 13.33% were diagnosed more than 5 years ago. The qualitative findings revealed six major themes. Realisation and Acceptance showed that caregivers noticed early behavioural issues and felt fear, sadness and helplessness upon diagnosis. Acceptance and Family Support highlighted how caregivers slowly accepted the condition with help, though some family members were unsupportive. Understanding the Disability included awareness of causes like birth complications and signs such as delayed speech and poor focus. Daily Challenges and Social Issues covered learning difficulties and stigma, leading to emotional strain. Knowledge and Experience of Treatment showed that regular therapy and follow-up led to minor but encouraging improvements. Care Needs and Psychological Burden reflected the need for support services and emotional toll on caregivers, who reported stress, exhaustion and anxiety about their child's future. Overall, the themes reflected both the challenges and coping efforts associated with caregiving. Before the intervention, 76.16% of caregivers experienced distress, whereas after the intervention, 38.84% of caregivers experienced distress. The reduction of 39.32% indicated the effectiveness of the study. The pretest, caregivers had a mean strain score of 10.05, whereas in the post-test, they had a mean score of 4.82. The difference of 5.23 was found to be large and statistically significant. This was confirmed using the paired t-test (Table 3 - see PDF). The association between caregivers' post-test level of distress scores and their demographic variables was analyzed. Caregivers aged 41-45 years and those belonging to extended families had a higher proportion of mild distress scores. Statistical significance was determined using the Chi-square test. The association between caregivers' post-test level of distress scores and children's demographic variables was also assessed. Caregivers of children with a third or higher birth order had a higher proportion of mild distress scores. Statistical significance was determined using the Chi-square test. The association between caregivers' post-test level of strain scores and their demographic variables was examined. Caregivers' age and family type were associated with a higher proportion of mild strain scores. Statistical significance was determined using the Chi-square test. The association between caregivers' post-test level of strain scores and children's demographic variables was also analyzed.

Caregivers with three or more children in the family had a higher proportion of low strain scores. Statistical significance was determined using the Chi-square test. The study revealed significant insights through integrated qualitative and quantitative findings. Qualitatively, six themes emerged, capturing caregivers' journey from realisation and acceptance of their child's condition to facing emotional and social challenges. Parents described early concerns about unusual behaviours and experienced fear, sadness and helplessness. Gradual acceptance occurred with family and professional support, though some relatives remained unsupportive. Caregivers demonstrated growing awareness of the disability's causes and symptoms and reported difficulties in education and societal stigma. They valued therapy and observed modest improvements, while also expressing emotional exhaustion and concern for their child's future. Quantitatively, the intervention proved effective: distress and strain levels significantly reduced post-intervention. High and very high distress levels dropped to moderate or low and mean scores for distress and strain declined markedly. Statistical tests confirmed the intervention's positive impact, aligning with caregivers' reported experiences coping improvements. Figure 1 (see PDF) shows the percentage of caregivers categorised by different age groups. This visual representation provides insight into the distribution of caregivers across various age ranges. Figure 2 (see PDF) illustrates the percentage of caregivers belonging to nuclear, joint, and extended families. This chart highlights how family structure influences the caregiving experience, with varying proportions represented across the three types of family structures. Figure 3 (see PDF) presents the overall percentage of caregivers in the study, giving a broader view of the caregiver population involved in the research. This data allows for an understanding of the general caregiver distribution in the study. Figure 4 (see PDF) depicts the percentage of caregivers based on family structure, further emphasising how different types of families (such as nuclear or extended) contribute to the caregiving role. It helps identify trends in caregiver support based on family type.

## Discussion:

The qualitative findings revealed six major themes including realization, acceptance, understanding disability, daily challenges, treatment experience and psychological burden among caregivers. These themes align with studies by Zainal *et al.* (2025) [12], emphasizing caregiving challenges and support needs. 30% of the primary caregivers are below 30 years of age, 16.67% are between 31-35 years, 33.33% fall in the 36-40 years range and 20% are aged 41-45 years.60% of the caregivers are female, while 40% are male. Among caregivers, 41.67% identify as Hindu, 25% as Christian, 15% as Muslim and 18.33% belong to other religions. Family types include 41.67% from nuclear families, 36.67% from joint families and 21.66% from extended families. Residential areas are distributed as 38.33% urban, 46.67% semiurban and 15% rural. Two-thirds (66.67%) of the caregivers are in consanguineous marriages, with 33.33% in non-consanguineous marriages. Educational qualifications show 13.33% with informal education, 26.67% with primary education, 20% completed higher secondary school, 23.33% are graduates and 16.67% hold professional qualifications.

Occupational status includes 8.33% homemakers, 25% unskilled workers, 18.33% clerks/shop owners/fathers, 28.34% semi-professionals and 20% professionals. Monthly family income distribution reveals 38.33% earn below ₹5,000, 25% earn between ₹5,000 and ₹10,000 and 36.67% earn more than ₹10,000.26.67% of the children are aged between 1-5 years, 20% are between 6-10 years and the majority, 53.33%, fall within the 11-15 years age group.51.67% of the children are male, while 48.33% are female.46.67% of the children are first-born, 25% are second-born and 28.33% are third-born or beyond.45% of the families have one child, 13.33% have two children and 41.67% have three or more children.10% of the children were diagnosed within the last 6 months to 1 year, 40% received their diagnosis between 1-3 years ago, 36.67% between 3-5 years ago and 13.33% were diagnosed more than 5 years ago. The pre-test scores showed that 16.67% of caregivers had high psychological distress, while 83.33% experienced very high distress. All caregivers reported high strain levels before intervention. These findings align with studies by Ramasubramanian (2019) [10], highlighting significant psychological burdens among caregivers of intellectually disabled children. Post-test results revealed a marked improvement following the nurse-led intervention, with 35% of caregivers experiencing low psychological distress and 65% reporting moderate distress-none had high or very high distress. Similarly, 73.33% had low strain and only 26.67% had higher strain. These findings align with studies by Kao *et al.* (2025) [13], both demonstrating that structured psychosocial interventions significantly reduce psychological distress and enhance caregiver well-being. The present study revealed a significant reduction in psychological distress and strain among primary caregivers post-intervention, with a fair positive correlation between both variables. This suggests that reducing distress also lowers strain, highlighting the importance of targeted mental health support for caregivers. The study found significant associations between caregivers' post-test distress and strain levels with demographic variables like age, family type and number of children.

## Conclusion:

The study highlights the importance of recognizing caregivers as essential partners in care and addressing their needs through targeted nursing strategies. The findings suggest that nurse-led interventions are an effective, sustainable, and low-cost approach to reducing psychological burden and improving caregiving quality. This calls for the inclusion of caregiver-centered interventions in clinical practice, alongside policy initiatives and future research on long-term support models for both caregivers and clients in intellectually disabled populations.
